# Secondary Metabolites Profiling of* Acinetobacter baumannii* Associated with Chili (*Capsicum annuum* L.) Leaves and Concentration Dependent Antioxidant and Prooxidant Properties

**DOI:** 10.1155/2019/6951927

**Published:** 2019-02-05

**Authors:** Tahmina Monowar, Md. Sayedur Rahman, Subhash J. Bhore, Gunasunderi Raju, Kathiresan V. Sathasivam

**Affiliations:** ^1^Unit of Microbiology, Faculty of Medicine, AIMST University, 08100 Bedong, Kedah, Malaysia; ^2^Department of Biotechnology, Faculty of Applied Sciences, AIMST University, 08100 Bedong, Kedah, Malaysia; ^3^Regional Director, Ministry of Information, Government of the People's Republic of Bangladesh, Bangladesh Betar, Gopalganj, Bangladesh; ^4^School of Distance Education, Universiti Sains Malaysia, 11800 Pulau Pinang, Malaysia

## Abstract

Secondary bioactive compounds of endophytes are inevitable biomolecules of therapeutical importance. In the present study, secondary metabolites profiling of an endophytic bacterial strain,* Acinetobacter baumannii,* were explored using GC-MS study. Presence of antioxidant substances and antioxidant properties in chloroform (CHL), diethyl ether (DEE), and ethyl acetate (EA) crude extracts of the endophytic bacteria were studied. Total phenolic content (TPC), total flavonoid content (TFC), total antioxidant capacity (TAC), 2,2-diphenyl-1-picrylhydrazyl (DPPH) radical scavenging activity, and ferrous ion chelating assay were evaluated. A total of 74 compounds were identified from the GC-MS analysis of the EA extract representing mostly alkane compounds followed by phenols, carboxylic acids, aromatic heterocyclic compounds, ketones, aromatic esters, aromatic benzenes, and alkenes. Among the two phenolic compounds, namely, phenol, 2,4-bis(1,1-dimethylethyl)- and phenol, 3,5-bis(1,1-dimethylethyl)-, the former was found in abundance (11.56%) while the latter was found in smaller quantity (0.14%). Moreover, the endophytic bacteria was found to possess a number of metal ions including Fe(II) and Cu(II) as 1307.13 ± 2.35 ppb and 42.38 ± 0.352 ppb, respectively. The extracts exhibited concentration dependent antioxidant and prooxidant properties at high and low concentrations, respectively. The presence of phenolic compounds and metal ions was believed to play an important role in the antioxidant and prooxidant potentials of the extracts. Further studies are suggested for exploring the untapped resource of endophytic bacteria for the development of novel therapeutic agents.

## 1. Introduction

Oxidative stress is the state of an imbalance in favour of the prooxidants and disfavouring the antioxidants, potentially leading to damage of all types of biological molecules, including DNA, lipids, proteins, and carbohydrates [1]. Thus, oxidative stress may lead to the development of chronic degenerative diseases like coronary heart disease, cancer, and aging. Antioxidants can delay, inhibit, or prevent the oxidative stress by scavenging or neutralizing free radicals or their actions [2]. Nowadays, genotoxicity of some synthetic antioxidants such as butylated hydroxyanisole (BHA) and butylated hydroxytoluene (BHT) has created much attention to explore new antioxidants from natural resources [3, 4]. Plant-derived polyphenols and flavonoids possess antioxidative, free radical scavenging, anti-inflammatory, hepatoprotective, anticancer, antiviral, and coronary heart disease prevention activities [2, 5].

Bacteria that colonize in the interior of plants including active and latent pathogens are considered as endophytic bacteria [6]. Endophytes are well known for producing diverse range of secondary metabolites. Different types of natural products including antibiotics, anticancer, antifungal, antiviral, insecticidal, immunosuppressant, and volatile organic compounds have been derived or produced from various endophytic bacteria [7]. There has been an increasing interest in endophytic bacteria from the last several years due to their capability to mimic and produce similar bioactive compounds of their respective host plants [8–10] as well as new bioactive compounds that are not present in host plants [8].

The application of beneficial endophytic bacteria has opened up new possibilities in the field of biotechnology. Over that last couple of years, various roles of endophytic bacteria have been reviewed by several authors [7, 11–14]. Endophytic bacteria have been reported to play a vital role in growth promotion, nutrient management, disease control, and biotic and abiotic stress tolerance in food and nonfood crop plants. Endophytic bacteria are also known to produce several enzymes like serine-type fibrinolytic enzymes [15], 1-aminocyclopropane-1-carboxylate (ACC) deaminase [16], exo-*β*-agarase [17], and indole-3-acetic acid, IAA [16]. Recent studies showed that* L*-asparaginase enzyme [18] and a quinoline alkaloid compound, camptothecin [19], produced from endophytic bacteria have potential anticancer properties. Therefore, endophytic bacteria represent a potential source for the discovery of new and novel compounds of medicinal importance.

Apart from producing functional metabolites, endophytic bacteria also produce chemicals having pharmaceutical functions. They have great prospects for broad spectrum utility in medicine, agriculture, and industry [20]. Recent studies suggest that endophytic bacteria exhibit antimicrobial activity [21–23], antioxidant activity [18, 21, 24, 25], and DNA damage protecting activity [26]. A levan-type exopolysaccharide (EPS) was isolated from endophytic bacteria,* Paenibacillus polymyxa* EJS-3 [27], which had been reported to exhibit both* in vitro* and* in vivo* antioxidant activity [27, 28]. The EPS isolated from endophytic bacterium,* Bacillus cereus* SZ1, has also been reported to exhibit antioxidant property [26].

Presently, less than 1% of endophytes are known [29] suggesting millions of endophytic microorganisms to be studied systematically. Until recently, there are only a few literature reports on the antioxidant properties of the diverse and varied endophytic bacteria of different host plants origin [30]. Many available methods are used to evaluate the antioxidant activity of bioactive compounds [31]. However, because of the complexity involved in the* in vivo* mechanisms of action, more than a single* in vitro* chemical method has been suggested to evaluate and compare the antioxidant properties of natural products [3]. Moreover, due to the involvement of multiple reaction characteristics and mechanisms, no single assay is capable of accurately reflecting all antioxidants in a mixed or complex system [32]. Therefore, three complementary test methods were used in the present study to evaluate the* in vitro* antioxidant properties of different solvent extracts of the experimental endophytic bacteria.

Although prooxidant agents are well known for their detrimental effects on human health, they are also used as therapeutic agents [33]. The use of prooxidant agents is emerging as an exciting strategy to selectively target tumour cells. Cancer cells produce high levels of reactive oxygen species (ROS) leading to increased basal oxidative stress. Plant secondary metabolites with prooxidant and anticancer activities can be used as cancer chemotherapeutic agents. Although many natural products are known to affect the redox state of the cell, most studies on these compounds have focused on their antioxidant activity instead of on their prooxidant properties [34]. ROS is regarded as the target of the anticancer agents in cancer therapy. Prooxidants as anticancer agents induce apoptosis in cancer cells through promoting ROS signalling pathways and weakening the antioxidant defence system of cancer cells [35]. Flavonoids display prooxidant activity modulating cell signalling which is thought to be directly proportional to the total number of hydroxyl groups [36]. Phenolic compounds act as prooxidant agents under certain conditions like high concentrations, high pH, and the presence of redox-active metals like copper [33].

Antioxidants are of two classes: primary or chain-breaking antioxidants (mainly acting by ROS/RNS scavenging) and secondary or preventive antioxidants (usually acting by transition metal ion chelation [37]). Metal ions form a complex with flavonoids abundantly present in the plant kingdom, which can perform as effective free radical and metal scavenger [38]. In contrast, secondary or preventive antioxidants act as the most important lipid prooxidant that may retard or prevent lipid oxidation. They accelerate lipid peroxidation by breaking down hydrogen and forming lipid peroxides by Fenton-type reactions [39]. For instance, transition metal ion [namely, Fe(II) or Cu(I)] chelator antioxidants may inhibit Fenton-type reactions that produce hydroxyl radicals, which may cause oxidative degradation of biological macromolecules such as lipids, proteins, and DNA [37, 39]. Iron and other transition metals (copper, chromium, cobalt, vanadium, cadmium, arsenic, and nickel) promote oxidation by acting as catalysts of free radical reactions. These redox-active transition metals transfer single electrons during changes in oxidation states. Chelation of metals by certain compounds decreases their prooxidant effect by reducing their redox potentials and stabilizing the oxidized form of the metal. Chelating compounds may also sterically hinder formation of the metal hydroperoxide complex [40]. Furthermore, transition metal ions can act as prooxidants by inducing Fenton reaction and Haber-Weiss reaction leading to generation of excessive ROS. Some of the popular and well known antioxidant flavonoids have been reported to act as prooxidant under the presence of transition metal ions [41]. Therefore, presence of any metal ion affecting the antioxidant activity of different extracts of the endophytic bacteria was evaluated in the present study.

Metals like iron, copper, manganese, and cobalt either containing enzyme or other chemical constituents can produce hydroxyl radical and superoxide radical anion from hydrogen peroxide (H_2_O_2_) via Fenton reaction to prevent or retard metal ion induced lipid oxidation are described as a better antioxidant. Nevertheless, selection of the suitable metal or nonmetal oxidizing agent is considered as a determinant to modify the measurement of the reducing power [42]. Transition metals are indispensable for organisms due to their chemical properties such as redox activity (Cu, Fe) and Lewis acid strength (Zn) [43]. The impact of transition metals including zinc, iron, and copper for controlling plant pathogen infection has been reviewed in literature [44]. To date, there are available reports on heavy metals tolerance efficiency of different microbes including endophytes [45, 46]. Until recently, there is no single report focusing on the concentration of transition metal ions in the endophytic microbes that may contribute to their metal chelating activities. Therefore, an effort was made in the present study to elucidate some transition metals concentration in the endophytic bacteria with a view to a better understanding of their antioxidant properties.

Recent literature reports reveal that plant-based natural compounds of* Brassica oleracea* L. var.* sabauda* [47], red fruits teas [48],* Salicornia herbacea* L. [49], vegetables and agricultural by-products [50], ascorbic and humic acids [51],* Raphanus sativus *var.* niger* [52], green tea [53], and eugenol [54] display both antioxidant and prooxidant behaviours under certain circumstances. Several factors are known to influence the antioxidant and/or prooxidant properties of natural compounds, such as reagent used, procedure followed, analytical time, quantification criterion, transition metal ions, H_2_O_2_, temperature, pH, concentration of the compounds, solubility and polarity of the sample, dissolved oxygen, size of the micelles formed, and synergistic or antagonistic interactions of chemicals in complex samples [50, 55–57]. In the present study, a first ever attempt of its kind was undertaken to explore the presence of transition metal ions in the endophytic bacteria. Moreover, metabolic profiling of the endophytic bacteria was carried out to discover the presence of available secondary metabolites responsible for antioxidant properties. The present study will open up a new window to explore the promising biotechnological application potentials of other endophytic bacteria.

## 2. Materials and Methods

### 2.1. Collection of Endophytic Bacteria

Endophytic bacterial strain of* Acinetobacter baumannii* (GenBank accession no. HQ670501) was collected from the Department of Biotechnology, AIMST University, which was previously isolated from chili (*Capsicum annuum* L.) leaves and was identified using 16S rRNA molecular biomarker [58].

### 2.2. Preparation of Crude Extract

Crude extracts were prepared following the methods described in literature [59] with some modifications. The endophytic bacterial strain of* A*.* baumannii* was inoculated in a 125 mL Erlenmeyer flask containing 25 mL nutrient broth (Hi Media, India) using a Rotary Incubator Shaker (Model: Innova 40, New Brunswick Scientific, USA) at 150 rpm, 37°C for 24 h. The seed culture was then transferred to 1.0 L Erlenmeyer flask containing 475 mL nutrient broth and was kept in the incubator shaker at 150 rpm, 37°C for 24 h. The culture with the cell and supernatant was extracted with organic solvent (1:1v/v) using chloroform (QREC (ASIA), Malaysia), diethyl ether (QREC (ASIA), Malaysia), and ethyl acetate (QREC (ASIA), Malaysia) separately. The crude extracts were recovered after evaporating the organic solvents using a rotary vacuum evaporator (Model: Yamato RE300, Yamato Scientific Co. Ltd., Tokyo, Japan). The dry weight of the crude extracts was measured using a digital weighing machine and dissolved in 1% dimethyl sulfoxide, DMSO (QREC (ASIA), Malaysia) with a final concentration of 10 mg/mL.

### 2.3. Determination of Antioxidant Compounds

#### 2.3.1. Total Phenolic Content

Total phenolic content was measured by following Folin-Ciocalteu's colorimetric method [60]. In brief, 0.1 mL of sample and 0.5 mL of Folin-Ciocalteu reagent (Sigma-Aldrich, USA) were mixed to 6.0 mL of double-distilled water. After 1 min, 1.5 mL of 20% Na_2_CO_3_ (Merck, Germany) was added and the total volume was made up to 10.0 mL with double-distilled water. The mixture was incubated for 2 h at 25°C. The absorbance was measured at 760 nm using a spectrophotometer (DU® 800 UV/Vis Spectrophotometer, Beckman Coulter, USA) against the blank solution containing all the reagents and the appropriate volume of the same solvent used for the sample. Gallic acid (R & M Chemicals, UK) was used as the control containing all the reaction agents except the sample. The amount of total phenolics present was expressed as *μ*g of gallic acid equivalent per mg of extract (*μ*g GAE/mg)

#### 2.3.2. Total Flavonoid Content

Total flavonoid content was measured by using the AlCl_3_ colorimetric method [61] with slight modifications. Rutin (HmbG Chemicals, Germany) was used to make the calibration curve. One milligram of rutin was dissolved in methanol (R & M Chemicals, UK) and then diluted to 62.5, 125, 250, 500, and 1000 *μ*g/mL. The diluted standard solutions (0.5 mL) were separately mixed with 1.5 mL of methanol, 0.1 mL of 10% AlCl_3_ (R & M Chemicals, UK), 0.1 mL of 1M potassium acetate (Sigma-Aldrich, USA), and 2.8 mL of distilled water. After incubation at room temperature for 30 min, the absorbance of the reaction mixture was measured at 415 nm using the UV-Vis spectrometer. The amount of 10% AlCl_3_ was substituted by the same amount of distilled water in blank. Similarly, 0.5 mL of sample solution was reacted with AlCl_3_ for determination of flavonoid content as described above. The TFC was expressed as *μ*g of rutin equivalent per mg of extract (*μ*g RE/mg).

### 2.4. *In Vitro* Antioxidant Assay

#### 2.4.1. Total Antioxidant Capacity

Total antioxidant capacity was measured by following the phosphomolybdenum method [62]. Sulfuric acid (Merck, Germany), sodium phosphate (Sigma-Aldrich, USA), and ammonium molybdate (Gem Chemicals, Malaysia) were used to prepare the reagent solution. In brief, an aliquot of 0.1 mL of sample solution was combined in an Eppendorf tube with 1.0 mL of reagent solution (0.6 M sulfuric acid, 28 mM sodium phosphate, and 4 mM ammonium molybdate). The tube was capped and incubated in a thermal block at 95°C for 90 min. The sample was allowed to cool to room temperature, and then the absorbance of the solution of each was measured at 695 nm against a blank. A typical blank solution containing 1.0 mL of reagent solution and the appropriate volume of the same solvent used for the sample was incubated under the same conditions. Ascorbic acid (HmbG Chemicals, Germany) was used as the control, and the TAC was expressed as *μ*g of ascorbic acid equivalent per mg of extract (*μ*g AAE/mg). The TAC was calculated using the following linear equation based on the calibration curve for ascorbic acid:

(1) $$\begin{array}{c} Y=0.0011X+0.0121\;\left(R^{2}=0.9963\right) \end{array}$$

where Y is absorbance at 695 nm and X is concentration of ascorbic acid (*μ*g/mL).

#### 2.4.2. DPPH Radical Scavenging Assay

The DPPH (2,2-diphenyl-1-picrylhydrazyl) radical scavenging activity was carried out by using DPPH (Merck, Germany) as a free radical [63]. In brief, an aliquot of 0.1 mL sample (conc. 62.5, 125, 250, 500, and 1000 *μ*g/mL) was mixed with 3.9 mL of a freshly prepared DPPH solution in methanol (25 mg/L). The mixture was vortexed vigorously and kept in the dark condition for 2 h at room temperature. Then the absorbance of the mixture was determined at 515 nm against the blank. DPPH solution without the sample was used as the control. Gallic acid and ascorbic acid were used as the standards. DPPH radical scavenging activity was expressed as efficient concentration, EC_50_ (mg/mL).

Moreover, the percentage of DPPH scavenging capacity was also measured following the Blois method [64], where the DPPH solution in methanol (DPPH solution without sample) was used as the control in the above experiment. The percentage inhibition activity was calculated using the following equation:

(2) DPPH  radical  scavenging  effect%  of  inhibition=Acontrol−AsampleAcontrol×100

where A_control_ is absorbance of the control reaction and A_sample_ is absorbance of the sample/standard.

#### 2.4.3. Ferrous Ion Chelating Assay

Ferrous ion chelating activity was estimated by following the method of Dinis and colleagues [65] with slight modifications. In brief, 0.05 mL of 2 mM FeCl_2_ (R & M Chemicals, UK) was added to 1 mL of different concentrations of the extracts (0.5, 1.0, 1.5, 2.0, and 2.5 mg/mL). The reaction was initiated by the addition of 0.2 mL of 5 mM ferrozine (HmbG Chemicals, Germany) solution. The mixture was vigorously shaken and left to stand at room temperature for 10 min. The absorbance of the solution was thereafter measured at 562 nm against the reagent blank. The control contained FeCl_2_ and ferrozine, with complex formation molecules. Na_2_-EDTA (Sigma-Aldrich, USA) was used as the positive control. The percentage inhibition of ferrozine-Fe^2+^ complex formation was calculated as below:

(3) $$\begin{array}{c} Inhibition\left(\;\%\;\right)=\left[\;\frac{\left(A_{control}-A_{sample}\right)}{A_{control}}\;\right]\times 100 \end{array}$$

where A_control_ is absorbance of the control reaction and A_sample_ is absorbance of the sample/standard.

### 2.5. Secondary Metabolites Profiling Using GC-MS

Only the EA extract of the endophytic bacteria was considered for GC-MS analysis based on the antioxidant study results. Agilent Technologies GC (7890A) equipped with a series injector (Model 7683B) and coupled with an Agilent Technologies inert MSD (5975 C) with Triple-Axis MSD was used for the analysis. HP-5MS (30 m x 0.25 mm) coated with 5% Phenyl Methyl Silox (0.25 *μ*m film) was used as the GC column. The scan frequency was 2.64 times per second and the mass range scanned was 30-800 amu. The carrier gas was helium (99.999%) at a constant flow rate of 1.0 mL/min. A split injection technique was used and injection port temperature was 250°C. The oven program was as follows: 50°C for 0 min, then 3°C /min to 230°C for 5 min, and 300°C for 70 min. Mass spectra recorded during the analysis were compared to spectra contained in the NIST Mass Spectral Library using NIST 05 Database [66]. The compounds tentatively identified refer to identities whose spectral data matched those found in NIST library workbook (https://webbook.nist.gov/chemistry/).

### 2.6. Analysis of Metal Ion Concentration Using ICP-MS

Inductively Coupled Plasma-Mass Spectrometry (ICP-MS) is one of the sensitive and accurate analytical techniques that determines the different trace and ultratrace elements in various biological and pharmaceutical samples [67]. In the present study, a Triple Quadrupole ICP-MS (NexION 300 ICP-MS connected with the Triple Quadrupole MS) with an S10 auto sampler (PerkinElmer Co., USA) was used for the analysis of different trace elements such as Na, Mg, Al, K, Ca, Cr, Fe, Ni, Cu, Zn, Cd, and Pb in the endophytic bacteria following the AOAC method [68]. A multielemental standard working solution of the elements (ICP multielement standard solution IV, Sigma-Aldrich, USA) was prepared by step-wise dilutions of monoelemental stock solutions (10,000 ppb). Mixed standards of five different concentrations (namely, 1 ppb, 5 ppb, 10 ppb, 20 ppb, and 100 ppb with the dilution factors 100, 20, 10, and 5, respectively) were used to prepare the standard calibration curve. The samples were diluted 1:10 in 18 MΩ deionized water (Merck, Germany) just prior to analysis. The spectral data of the Quadrupole Mass Spectrometer were processed with the Chromera software. The tests were done in triplicate and the results were expressed as mean ± SD.

### 2.7. Statistical Data Analysis

Each experiment was performed in triplicate independently and the results were presented as mean ± SD. Tukey's HSD test was performed using IBM SPSS Statistics for Windows, Version 22.0 (IBM Corp., Armonk, NY, USA), to elucidate the significant difference among the mean values of the experimental groups. Differences were considered statistically significant at* P *< 0.05 level.

## 3. Results and Discussion

Phenolics [2] and flavonoids [5] are responsible for exhibiting antioxidant property of chemical compounds. Hence, presence of antioxidant compounds such as total phenolic content and total flavonoid content in three different solvent extracts, secondary metabolites, and metal ions of the endophytic bacteria was evaluated in the present study with a view to evaluate their biotechnological prospects in future pharmaceutical applications.

### 3.1. Antioxidant Compounds

#### 3.1.1. Total Phenolic Content

In the present study, total phenolic content (TPC) of different solvent extracts of the experimental endophytic bacteria is shown in Table 1. The EA extract exhibited the highest amount of TPC (967.78 ± 34.65 *μ*g GAE/mg) while the CHL extract exhibited the lowest amount of TPC (254.44 ± 5.36 *μ*g GAE/mg). As an extraction solvent, EA is selective in extracting low-molecular-weight phenolic compounds and high molecular weight polyphenols [69]. Besides, EA allows the highest phenolic content while selectively removing nonphenolic compounds from the extractible matters [70]. The presence of the highest TPC value in the EA extract of the endophytic bacteria,* A. baumannii* compared to the CHL and DEE extracts in the present study is consistent with the earlier studies for evaluating TPC from endophytes as reported in literature [71, 72].

There was observed statistically significant difference (*P* < 0.05) among TPC of different solvent extracts suggesting the fact that TPC is dependent on the extraction solvents [72, 73]. The amount of TPC in the aqueous extract of ten endophytic bacteria isolated from eight different ethnomedicinal plants collected from Northeast India was reported to be in the range of 10.5 ± 0.01 to 16.0 ± 0.005 mg GAE/g of extract [18]. The concentration of TPC in the CHL fraction of the endophytic* Lactobacillus* sp. isolated from the leaf tissues of plant,* Adhatoda beddomei* has been reported to be 0.67 mg/mL [24]. However, the amount of TPC in the present study was found higher than those of the previous studies mentioned above. Therefore, the endophytic bacterial strains of* A. baumannii* in the present study could be a promising resource for natural phenolic compounds.

#### 3.1.2. Total Flavonoid Content

Total flavonoid content (TFC) in different solvent extracts of the endophytic bacteria is shown in the Table 1. Solvent dependent variation in the amount of TFC was observed in the present study in the range of 223.33 ± 33.33 *μ*g RE/mg (CHL extract) to 615.00±30.05 *μ*g RE/mg (EA extract). Solvent dependent TFC variation found in the present study is in well agreement with the earlier studies [74], where the authors reported TFC in methanol and acetone extracts of the fruits of* Indigofera caerulea* as 102.10 ± 2.3 mg QE/100 g and 3.84 ± 1.4 mg QE/100 g, respectively. Furthermore, studies with the endophytic fungus,* Aspergillus fumigatus,* MD-R-1 had been reported to exhibit the highest TFC (356.89 mg RE/g extract) in the EA extract, while the lowest TFC (42.58 mg RE/g extract) was reported in the water extract [72]. Besides, TFC of different fungal extracts has been reported by several authors in the range of 23.90 ± 0.001 to 11.92 ± 0.001 mg RE/g [75] and 8.27 ± 0.12 to 4.56 ± 0.08 mg RE/g [8]. The TFC value observed in the present study was higher than those of the previous studies reported in literature [72, 75]. It is therefore suggested that the endophytic* A*.* baumannii* can be a potential source of natural flavonoid compounds.

#### 3.1.3. Secondary Metabolites

**Figure 1:**
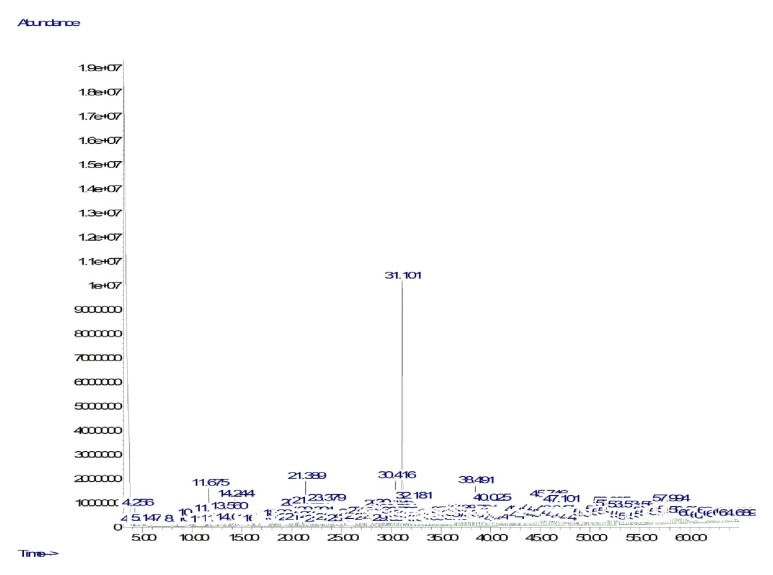
GC-MS profile of secondary metabolites from the endophytic* A*.* baumannii*.

In the present study, the EA extract of the endophytic bacteria was found to produce better extractables resulting in exhibiting better phytochemical properties (Table 1). For this reason, secondary metabolites in the EA extract of the endophytic bacteria were evaluated using GC-MS analysis. The GC-MS profile of the EA extract of the* A*.* baumannii* showed a total of 192 hits from which 74 compounds were identified (Table S1), and the GC-MS profile of the secondary metabolites is shown in Figure 1. The compounds were mostly representing alkanes (81.68%) followed by phenols (11.70%), carboxylic acids (2.45%), aromatic heterocyclics (1.56%), ketones (1.38%), aromatic esters (0.22%), aromatic benzenes (0.50%), and alkenes (0.05%). However, phenol, 2,4-bis(1,1-dimethylethyl)- was the only compound that was found as the single most abundant compound (11.56%) while phenol, 3,5-bis(1,1-dimethylethyl)- was found in less quantity (0.14%). The presence of these two phenolic compounds in the endophytic* A. baumannii* could be able to represent antioxidant properties [2].

#### 3.1.4. Metal Ions

In the present study, concentration of a total of 12 metals such as Na, Mg, Al, K, Ca, Cr, Fe, Ni, Cu, Zn, Cd, and Pb in the endophytic bacteria was evaluated using ICP-MS. The results are given in the Table 2. It was revealed from the present study that the abundance of the experimental transition metals ions in the endophytic bacteria was found in the order of Mg^2+^ > Ca^2+^ > Fe^2+^ > Zn^2+^ > Al^3+^ > Cr^2+^ > Ni^2+^ > Cu^2+^ > Pb^2+^ > Cd^2+^. Variation in the metal concentration among the endophytic bacteria might be associated with the plant metal concentration. These metal ions can contribute to the direct promotion of plant growth through nutrients acquisition or modulating hormonal levels with plants [76]. However, to the best of our knowledge, there is no available literature report except the present one dealing with measuring transition metal concentration in the endophytic microorganisms.

Microorganisms produce various minerals including metals, sulphides, and oxides. These minerals as nanosized materials generally exist in inter-, intra-, or extracellular spaces of the organisms. A detailed account on the process of microbial mineralization process has been described in literature [77]. Recently it has been reported that the bacterial species of* Lysinibacillus xylanilyticus* and* Lysinibacillus macrolides* are able to produce elemental selenite nanoparticles under aerobic conditions through either an intra- or extracellular reduction process [46]. The occurrence of the transition metals observed in the present study might be resulting from the accumulation of nanosized metals through the mineralization process of the endophytic bacteria.

### 3.2. *In Vitro* Antioxidant Assay

There are a number of* in vitro* assay methods for evaluating antioxidant properties of compounds. In the present study, total antioxidant capacity, DPPH radical scavenging activity, and ferrous ion chelating activity were carried out to assess the antioxidant potentials of three different solvent extracts of the endophytic* A*.* baumannii*.

#### 3.2.1. Total Antioxidant Capacity

The TAC values in different solvent extracts of the endophytic bacteria in the present study are shown in Table 1. The TAC values (*μ*g AAE/mg) were found in the range of 564.20 ± 1.72 (CHL extract) to 673.59 ± 1.20 (EA extract). There were observed no statistically significant difference (*P* < 0.05) in the TAC value between the CHL and DEE extracts. It was revealed from the present study that the composition of solvent might have a significant effect on the TAC values of the endophytic bacteria. However, all the extracts of the endophytic bacteria exhibited reducing capacity of Mo(VI) to Mo(V). It has been reported that the antioxidant effect is concomitant with the development of reductones [78], and reductones are the terminators of free radical chain reactions [37]. Thus, the TAC of different extracts of the endophytic bacteria may be related to their reductive activity.

#### 3.2.2. DPPH Radical Scavenging Activity

In the present study, the DPPH radical scavenging activity of the endophytic bacteria revealed the presence of antioxidant potential in a concentration dependent manner (Table 3). The CHL extract exhibited negative antioxidant (i.e., prooxidant) activity at all the concentration levels under study. The EA extract exhibited the highest percentage of inhibition with lower IC_50_ value, which was not significantly different (*P* < 0.05) with that of the DEE extract, especially at the higher level of concentrations (i.e., 500–1000 *μ*g/mL of the extracts). Furthermore, the DEE and EA extracts of* A. baumannii* were found to be able to reduce the stable free DPPH radicals to the yellow coloured diphenylpicrylhydrazyl at the higher level of concentrations (500–1000 *μ*g/mL). The results suggest that the extracts of the endophytic bacteria contain some active constituents that are capable of donating hydrogen to a free radical in order to remove odd electron from the lipid substrate which is responsible for free radical scavenging activity. However, none of the extracts of the endophytic bacteria were found as effective DPPH radical scavengers as those of the positive controls, gallic acid, and ascorbic acid.

Dose-dependent antioxidant/prooxidant activity was observed for different extracts of the endophytic bacteria in the present study, which corroborates with the earlier works reported in literature [79]. The authors studied DPPH radical scavenging activity with the EA extract of endophytic* Streptomyces* strain Eri12 isolated from* Rhizoma curcumae* and reported that the extracts exhibited dose-dependent antioxidant activity with IC_50_ value of 842.18 ± 161.24 *μ*g/mL [79]. Another antioxidant study with the EA extracts of four endophytic bacteria,* Pseudomonas hibiscicola *(ALR-22),* Macrococcus caseolyticus *(ALS-1),* Enterobacter ludwigii *(ALS-2), and* Pseudomonas nitroreducens *(ALR-5), collected from* Aloe vera *plants in Malaysia, has been reported to exhibit higher DPPH radical scavenging activities than the present study, ranging from 79% to 88%. Among them,* P. hibiscicola *(ALR-22) and* M. caseolyticus *(ALS-1) have been reported to exhibit the highest DPPH radical scavenging activities (88%), while the three isolates,* Sphingobacterium multivorum *(ALS-5),* Pseudomonas mosselii *(ALS6), and* Sphingobacterium siyangense *(ALS-4), have been reported to exhibit a lower DPPH radical scavenging activity with 15%, 25%, and 28% of inhibition, respectively. DPPH radical scavenging activity with the IC_50_ values (*μ*g/mL) ranging from 15.1 ± 0.05 to 18.6 ± 0.1 has been reported with* Pseudomonas hibiscicola (*ALR-22),* Macrococcus caseolyticus (*ALS-1), and* Enterobacter cancerogenus (*ALR-18), respectively [21], which were lower than the present study. A recent study shows that EA extract of endophytic actinomycete* Streptomyces *sp. A0916 collected from* Polygonum cuspidatum* exhibits the highest level of DPPH radical scavenging activity (93.2% inhibition) comparable to the standard, ascorbic acid (93.8% inhibition) at 128 *μ*g/mL [25]. On the contrary, a strong DPPH radical scavenging activity has been reported for the CHL extract of the endophytic bacteria,* Lactobacillus* sp. with IC_50_ value of 35 *μ*g/mL [24]. Hence, the DPPH radical scavenging activity of the extracts in the present study is lower than those of the* Streptomyces *sp. A0916 [25] and* Lactobacillus* sp. [24]. However, the dose-dependent and solvent dependent variation in the DPPH radical scavenging activity as observed in the present study are consistent with the previous antioxidant activities of different solvent extracts of an endophytic fungus,* Phomopsis liquidambari* QH4, isolated from* Artemisia annua* [73], and endophytic* Aspergillus *sp. JPY1 [80].

Phenolics and flavonoids are secondary plant metabolites that are ubiquitously observed among the plant-associated endophytic bacteria. The presence of such metabolites enhances the level of antioxidant [81]. The extraction of bioactive compounds from a sample is directly related to the compatibility of the compounds with the solvents [82]. Accordingly, it was observed in the present study that solvents with different polarity significantly altered the DPPH radical scavenging activity. In addition to that, all the extracts of the endophytic bacteria in the present study exhibited prooxidant activity at some concentration levels (Table 3). The CHL extract of the endophytic* A. baumannii* exhibited prooxidant activity up to the highest level of concentration (1000 *μ*g/mL) studied in the present study. On the other hand, the DEE and EA extracts exhibited prooxidant activity up to the concentration level of 250 *μ*g/mL. The present findings suggest that the prooxidant effects of the extracts are concentration dependent as most of these extracts exhibited antioxidant effects at higher level of concentration (Table 3). Moreover, it is obvious from the present study that the experimental endophytic bacteria contain diverse secondary metabolites including phenolic compounds (Table S1). The presence of a diverse range of antioxidant compounds (Tables 1 and S1) as well as metal ions (Table 2) along with their synergistic and/or antagonistic effects might have resulted in both the antioxidant and prooxidant activities of the endophytic bacteria at different concentrations in the present study.

Both the natural phenolic and synthetic antioxidants have been reported to show prooxidant activity at low concentrations by several authors. For instance, natural phenolic compounds [83], ascorbic acid [84], aloin from the medicinal plant,* Aloe* sp. [85], the endophytic fungi* Entrophospora infrequens* isolated from the medicinal plant* Nothapodytes foetida* in lipid phase [86], and red grape peels [50] have been reported to exhibit prooxidant activity at low concentrations. Furthermore, concentration dependent antioxidant and prooxidant behaviour of several natural chemopreventive agents have also been attributed in literature, such as quercetin [87], *β*-carotene and* N*-acetylcysteine [88], vitamin C [88, 89], and curcumin [90]. In the present study, dose-dependent antioxidant and prooxidant behaviours of the endophytic bacterial extracts are prominent, which are consistent with the previous findings as mentioned above.

Mixtures of different phenolic compounds can produce synergistic or antagonistic prooxidant effects [91]. Electrochemical behaviour of natural phenolic compounds also plays a vital role in determining their antioxidant and/or prooxidant properties. For instance, phenolic compounds with low anodic oxidation potentials, Epa < 0.45 V (namely, salicylic acid,* m-*hydroxybenzoic acid,* p-*hydroxybenzoic acid, vanillic acid, syringic acid,* O-*coumaric acid,* m-*coumaric acid, and* p-*coumaric acid), display antioxidant activity, while compounds with high anodic oxidation potentials, Epa > 0.45 V values (namely, protocatechuic acid, caffeic acid, quercetin and rutin), act as prooxidants [92]. In the present study, the dual nature of the extracts might be due to an antagonistic interaction with higher oxidation potential phenolic compounds present in the extract which might have simultaneously displayed antioxidant and prooxidant activity [93]. The nature of extraction solvents also affects antioxidant and/or prooxidant activity of the extracts [93]. In the present study, it is assumed that a better extraction of phenolic compounds that can be oxidized and reduced in that range might have allowed the extracts of* A. baumannii* presenting both of their antioxidant and prooxidant activities [93]. Therefore, dose selection is the suggestive of an important factor in the application of antioxidants.

#### 3.2.3. Ferrous Ion Chelating Assay

The results of the ferrous ion chelating activity of different solvent extracts of the experimental endophytic bacteria are shown in Table 4. The results show that the EA extract displayed the highest percentage of inhibition (0.28 ± 0.04%) followed by the DEE and CHL extracts as -15.23 ± 0.14% and -21.89 ± 0.221%, respectively, at the highest level of concentration (2.5 mg/mL). The highest percentage inhibition at 2500 *μ*g/mL concentration was significantly lower than that of the standard chelating agent, Na_2_-EDTA (98.27 ± 0.12%) at 250 *μ*g/mL concentration level, which was about 500 times lower with respect to IC_50_ value. A higher percentage of inhibition than the present study has been reported for the exopolysaccharide extract of the endophytic bacterium* Paenibacillus polymyxa* as 92.4% at 1 mg/mL concentration [28], the ethanol extract of the endophytic bacterium* Streptomyces *sp. MOE6 as 92.0 ± 0.1% at 2 mg/mL concentration [94], and methanol extract of endophytic* Aspergillus *sp. JPY1 as 41.5% at 1 mg/mL concentration [80].

There was observed a marked difference between DPPH scavenging and ferrous ion chelating activities with respect to IC_50_ values. Lower ferrous ion chelating activity (higher IC_50_ values) than DPPH scavenging activity (lower IC_50_ values) was observed with all the extracts. Similar findings have been reported for the ethanolic leaf extracts of* Newbouldia laevis* [95], and various solvent extracts of* Sonchus asper* (L.) Hill. [96]. Furthermore, in terms of IC_50_ value, all the extracts of the endophytic bacteria in the present study were found to exhibit superior metal chelating activity than the ethanol extracts of the endophytic bacteria* Phialophora* Rct45 as 35.95 ± 0.050 mg/mL,* Lachnum* Rac76 as 28.89 ± 0.060 mg/mL,* Penicillium* Rct63 as 18.22 ± 0.069 mg/mL,* Dothideomycetes* Rsc57 as 12.06 ± 0.020 mg/mL [75], the culture supernatants of the endophytic bacteria* Citrobacter youngae* MEB5 as >60 mg/mL,* Bacillus *sp. F21 as >60 mg/mL,* Bacillus mycoides *M31 as >60 mg/mL,* Bacillus methylotrophicus *PotA as 13.25 ± 0.015 mg/mL,* Pseudomonas baetica *ENIB7 as 11.5 ± 0.004 mg/mL, and* Herminiimonas saxobsidens *AA as 11 ± 0.01 mg/mL [30]. However, all the extracts of the endophytic bacteria in the present study were found to be capable of chelating Fe^2+^ ions at some variable levels in dose-dependent manner. The present findings corroborate the earlier studies with the endophytic* Phomopsis liquidambari* QH4 [73] and endophytic* Aspergillus *sp. JPY1 [80].

Antioxidant potential of natural compounds depends on their chemical structures, especially the number and position of hydroxyl groups relative to the carboxyl functional group [90]. Easily oxidizable small phenolics such as quercetin and gallic acid possess prooxidant activity, while high molecular weight phenolics such as condensed and hydrolysable tannins have little or no prooxidant activity [97]. Phenolic and flavonoid antioxidants behave like prooxidants under certain conditions that favour their autoxidation, such as high pH, high concentrations of transition metal ions (namely, iron, copper, and zinc), presence of oxygen molecules/redox status, nature and concentration of the phenolic/flavonoid compounds under study and cell type [39, 53, 90]. It is therefore, not surprising to note in the present study the fact that over the concentration range of 500–1000 *μ*g/mL, DEE and EA extracts of the endophytic bacteria exhibited antioxidant activity in the DPPH assay while the same extracts exhibited prooxidant (i.e., negative percentage of inhibition) in the ferrous ion chelating assay. This phenomenon might be associated with the presence of high concentrations of Fe(II) and Cu(II) ions in the endophyte under the experimental conditions. Similar characteristics on the assay and concentration dependent antioxidant/prooxidant properties of some brown algal species are well documented. It has been reported that the dichloromethane: methanol (1:1, v:v) crude extracts of two brown algae,* Desmarestia ligulata* and* Dictyota dichotoma,* display prooxidant activities (-28.11±2.61 to -41.94 ± 0.71% and -24.17 ± 1.72% to -39.17 ± 1.77% at the concentrations ranging from 50 mg/L to 500 mg/L, respectively) with the *β*-carotene–linoleic acid system, but antioxidant activities with the DPPH radical scavenging and reducing assays [98]. So, it can be concluded that the antioxidant and prooxidant effects are dependent on concentrations and the methods applied to measure them. It is therefore suggested that the endophytic bacteria* A*.* baumannii* can be a promising natural metal chelating agents that may be used to prevent or retard metal ion induced lipid oxidation [99] and to treat Parkinson Disease [39]. Further exploration of novel endophytic bacterial species from diverse range of environment is the suggestive to identifying the best candidate as the natural source of antioxidant and prooxidant compounds.

## 4. Conclusion

Antioxidant and prooxidant properties of an endophytic bacterial strain,* A*.* baumannii,* were demonstrated in the present study using three different solvent extracts. The study revealed that the endophytic* A*.* baumannii* possesses a variety of secondary metabolites and transition metal ions that may play a significant role in presenting antioxidant and prooxidant properties in the dose and assay dependent manner. Phenolic compound was observed as the single major secondary metabolite in the EA extract of the experimental species. The endophytic species also possesses metal ions and hence can be regarded as the promising source of natural prooxidant agent. The study suggests the potential of the endophytic bacteria as the natural agents against free radical associated and metal ion induced oxidative damage. To the best of our knowledge, this is the first ever study investigating the antioxidant and prooxidant activities, and the abundance of secondary metabolites and metal ions in the endophytic bacteria. Further studies are strongly suggested for a complete understanding of their use as antioxidant, prooxidant, anti-cancer, and metal detoxifying agents.

## Figures and Tables

**Table 1:** TPC, TFC, and TAC of different solvent extracts of the endophytic *A*. *baumannii*.

Endophytic bacteria	Solvent	TPC (*µ*g GAE/mg ext)	TFC (*µ*g RE/mg ext)	TAC (*µ*g AAE/mg ext)
*A. baumannii*	CHL	254.44±5.36^c^	223.33 ± 33.33^b^	564.20 ± 1.72^b^
	DEE	750.56±11.82^b^	576.11 ± 17.35^a^	568.29 ± 4.67^b^
	EA	967.78±34.65^a^	615.00 ± 30.05^a^	673.59 ± 1.20^a^

Results are shown as mean±SD (n=3).

GAE: gallic acid equivalent, RE: rutin equivalent, and AAE: ascorbic acid equivalent.

Significant differences among the mean values of each column determined by Tukey's HSD test (*P* < 0.05) are indicated by different letters (a–c).

**Table 2:** Concentration of metal ions in the endophytic *A*. *baumannii*.

Metal ion	Concentration (ppb)	LOD	*r* ^*∗*^
Na^+^	ND	< 20.034	0.999
Mg^2+^	10365.72±158.30^a^	< 3.661	0.999
Al^3+^	200.68±2.73^e^	< 0.383	0.999
K^+^	ND	< 20.756	0.999
Ca^2+^	4507.02±61.78^b^	< 1.420	0.999
Cr^2+^	84.33±0.54^f^	< 0.059	0.999
Fe^2+^	1307.13±2.35^c^	< 1.162	0.998
Ni^2+^	48.74±0.10^g^	< 0.052	0.999
Cu^2+^	42.38±0.352^h^	< 0.045	0.999
Zn^2+^	304.10±2.61^b^	< 0.205	0.999
Cd^2+^	0.83±0.01^j^	< 0.023	0.999
Pb^2+^	2.60±0.01^i^	< 0.024	0.999

Results are shown as mean ± SD (n=3).

LOD: limit of detection; ND: not detected.

Significant differences among the mean values of each column determined by Tukey's HSD test (*P* < 0.05) are indicated by different letters (a–j).

^*∗*^Correlation coefficient of the calibration curve.

**Table 3:** DPPH radical scavenging activity of different solvent extracts of the endophytic *A*. *baumannii*.

Endophytic bacteria	Solvent	% Inhibition					EC_50_ (*μ*g/mL of extract)
		62.5 *μ*g/mL	125 *μ*g/mL	250 *μ*g/mL	500 *μ*g/mL	1000 *μ*g/mL	
*baumannii*	CHL	-18.87±0.28^c^	-17.63±0.50^c^	-16.39±0.28^c^	-12.12±0.14^b^	-5.37±0.28^b^	5626.28±112.20^b^
	DEE	-2.30±0.21^a^	-1.42±0.29^a^	-0.09±0.21^a^	1.84±0.08^a^	6.70±0.21^a^	4848.87±74.87^a^
	EA	-4.36±0.08^b^	-2.66±0.08^b^	-1.10±0.14^b^	1.88±0.21^a^	6.80±0.29^a^	4738.51±101.81^a^

Standard							

Gallic Acid		41.51±0.16	45.22±0.08	52.66±0.52	68.18±0.01	94.17±0.32	204.38±1.78

Ascorbic Acid		30.85±0.24	35.40±0.41	45.50±0.42	63.50±0.55	96.42±0.01	325.44±2.06

Results are shown as mean±SD (n=3).

Significant differences among the mean values of each column determined by Tukey's HSD test (*P* < 0.05) are indicated by different letters (a–c).

**Table 4:** Ferrous ion chelating activity of different solvent extracts of the endophytic *A*. *baumannii*.

Endophytic bacteria	Solvent	% Inhibition					IC_50_ (*μ*g/mL)
		500 *μ*g/mL	1000 *μ*g/mL	1500 *μ*g/mL	2000 *μ*g/mL	2500 *μ*g/mL	
*A. baumannii *	CHL	-41.84±0.54^c^	-37.46±0.32^c^	-32.10±0.21^c^	-28.26±0.11^c^	-21.89±0.221^c^	9871.34±172.42^a^
	DEE	-38.12±0.23^b^	-32.42±0.12^b^	-27.73±0.23^b^	-21.58±0.11^b^	-15.23±0.14^a^	9411.29±71.55^b^
	EA	-14.39±0.11^a^	-11.15±0.13^a^	-8.01±0.07^a^	-4.12±0.11^a^	0.28±0.04^a^	8318.68±57.07^c^

Standard							

Na_2_-EDTA		98.27 ± 0.12% at 250 *μ*g/mL conc.					15.76±0.03

Results are shown as mean±SD (n=3).

Significant differences among the mean values of each column determined by Tukey's HSD test (*P* < 0.05) are indicated by different letters (a–c).

## Data Availability

The data used to support the findings of this study are included within the article.

## Supplementary Materials

Supplementary Materials Table S1: secondary metabolites of the EA extract of the endophytic* A*.* baumannii*.
