# Supplementary material for: Secondary Metabolites Profiling of Acinetobacter baumannii Associated with Chili (Capsicum annuum L.) Leaves and Concentration Dependent Antioxidant and Prooxidant Properties
Source: Biomed Res Int. 2019 Feb 5;2019:6951927. doi: 10.1155/2019/6951927 (PMC6379878; doi:10.1155/2019/6951927)
Supplement: Supplementary Materials — Table S1: secondary metabolites of the EA extract of the endophytic A. baumannii. [file 6951927.f1.docx]

Table S1: Secondary metabolites of the ethyl acetate extract of the endophytic *A*. *baumannii*.

| Compound no | RT (min) | Area (%) | Hit Name | Quality | Mol. Formula | Mol. Weight |
| --- | --- | --- | --- | --- | --- | --- |
| 1 | 4.133 | 0.050 | Hexane, 2,3,5-trimethyl- | 72 | C_9_H_20_ | 128.26 |
| 2 | 4.251 | 0.513 | Heptane, 2,4-dimethyl- | 70 | C_9_H_20_ | 128.26 |
| 3 | 5.145 | 0.076 | Octane, 4-methyl- | 92 | C_9_H_20_ | 128.26 |
| 4 | 8.519 | 0.116 | Decane | 53 | C_10_H_22_ | 142.28 |
| 5 | 9.919 | 0.224 | Sulfurous acid, butyl hexyl ester | 47 | C_10_H_22_O_3_S | 222.34 |
| 6 | 10.138 | 0.096 | Nonane, 2,5-dimethyl- | 86 | C_11_H_24_ | 156.31 |
| 7 | 10.257 | 0.305 | Decane, 4-methyl- | 90 | C_11_H_24_ | 156.31 |
| 8 | 11.370 | 0.102 | Hexadecane, 2,6,10,14-tetramethyl- | 64 | C_20_H_42_ | 282.55 |
| 9 | 11.522 | 0.090 | Nonane, 1-iodo- | 72 | C_9_H_19_I | 254.15 |
| 10 | 11.674 | 1.453 | Undecane, 4,7-dimethyl- | 78 | C_13_H_28_ | 184.36 |
| 11 | 11.893 | 0.478 | Hexadecane, 7,9-dimethyl- | 78 | C_18_H_38_ | 254.50 |
| 12 | 12.585 | 0.144 | Hexadecane, 2,6,10,14-tetramethyl- | 50 | C_20_H_42_ | 282.55 |
| 13 | 13.242 | 0.137 | Hexane, 3,3-dimethyl- | 80 | C_8_H_18_ | 114.23 |
| 14 | 13.563 | 0.734 | Undecane, 4,7-dimethyl- | 78 | C_13_H_28_ | 184.36 |
| 15 | 13.799 | 0.250 | Eicosane | 81 | C_20_H_42_ | 282.55 |
| 16 | 14.086 | 0.254 | 1,3-Cyclopentadiene, 1,2,3,4-tetramethyl-5-methylene- | 78 | C_10_H_14_ | 134.22 |
| 17 | 14.238 | 1.170 | 2-Cyclohexen-1-one, 3,5,5-trimethyl- | 87 | C_9_H_14_O | 138.21 |
| 18 | 16.093 | 0.183 | Silane, cyclohexyldimethoxymethyl- | 87 | C_9_H_20_O_2_Si | 188.34 |
| 19 | 16.245 | 0.176 | Undecane, 2-methyl- | 86 | C_12_H_26_ | 170.33 |
| 20 | 16.785 | 0.223 | Undecane, 3,6-dimethyl- | 90 | C_13_H_28_ | 184.36 |
| 21 | 17.848 | 0.209 | Dodecane | 93 | C_12_H_26_ | 170.33 |
| 22 | 18.067 | 0.321 | Eicosane | 93 | C_20_H_42_ | 282.55 |
| 23 | 18.32 | 0.121 | Undecane, 2,4-dimethyl- | 87 | C_13_H_28_ | 184.36 |
| 24 | 18.472 | 0.395 | Undecane, 3,6-dimethyl- | 93 | C_13_H_28_ | 184.36 |
| 25 | 18.708 | 0.198 | 1H-1,2,4-Triazole | 27 | C_2_H_3_N_3_ | 69.07 |
| 26 | 18.809 | 0.335 | Dodecane, 4-methyl- | 93 | C_13_H_28_ | 184.36 |
| 27 | 19.788 | 0.271 | Decane, 2,4,6-trimethyl- | 80 | C_13_H_28_ | 184.36 |
| 28 | 20.041 | 0.253 | Tetradecane | 90 | C_14_H_30_ | 198.39 |
| 29 | 20.277 | 0.482 | Dodecane, 4,6-dimethyl- | 96 | C_14_H_30_ | 198.39 |
| 30 | 20.446 | 0.210 | Dodecane, 4-methyl- | 93 | C_13_H_28_ | 184.36 |
| 31 | 20.665 | 0.832 | 2,4-Dimethyldodecane | 80 | C_14_H_30_ | 198.39 |
| 32 | 21.087 | 0.178 | Pentacosane | 83 | C_25_H_52_ | 352.68 |
| 33 | 21.391 | 1.927 | Dodecane, 2,6,11-trimethyl- | 81 | C_15_H_32_ | 212.41 |
| 34 | 21.829 | 1.364 | Indole | 95 | C_8_H_7_N | 117.15 |
| 35 | 21.998 | 0.477 | Tridecane, 1-iodo- | 86 | C_13_H_27_I | 310.26 |
| 36 | 22.318 | 0.523 | Tridecane, 1-iodo- | 86 | C_13_H_27_I | 310.26 |
| 37 | 22.504 | 0.178 | Pyridine-3-carboxamide, oxime, N-(2-trifluoromethylphenyl)- | 56 | C_13_H_10_F_3_N_3_O | 281.24 |
| 38 | 22.605 | 0.265 | Hexane, 2,3,4-trimethyl- | 58 | C_9_H_20_ | 128.26 |
| 39 | 23.094 | 0.111 | 1-Iodo-2-methylundecane | 59 | C_12_H_25_I | 296.40 |
| 40 | 23.381 | 0.955 | Dodecane, 2,6,11-trimethyl- | 87 | C_15_H_32_ | 212.41 |
| 41 | 23.769 | 0.421 | Dodecane, 2,6,11-trimethyl- | 81 | C_15_H_32_ | 212.41 |
| 42 | 24.090 | 0.143 | Eicosane | 81 | C_20_H_42_ | 282.55 |
| 43 | 24.191 | 0.173 | Dodecane, 2,6,11-trimethyl- | 74 | C_15_H_32_ | 212.41 |
| 44 | 24.967 | 0.167 | Eicosane | 96 | C_20_H_42_ | 282.55 |
| 45 | 25.338 | 0.142 | Eicosane | 92 | C_20_H_42_ | 282.55 |
| 46 | 26.485 | 0.373 | Tetradecane | 95 | C_14_H_30_ | 198.39 |
| 47 | 26.873 | 0.251 | Pentadecane | 87 | C_15_H_32_ | 212.42 |
| 48 | 27.008 | 0.237 | Undecane | 83 | C_11_H_24_ | 156.31 |
| 49 | 27.076 | 0.293 | Eicosane | 91 | C_20_H_42_ | 282.55 |
| 50 | 27.396 | 0.529 | Tridecane, 1-iodo- | 83 | C_13_H_27_I | 310.26 |
| 51 | 28.088 | 0.319 | Eicosane | 93 | C_20_H_42_ | 282.55 |
| 52 | 28.661 | 0.737 | Pentadecane | 90 | C_15_H_32_ | 212.42 |
| 53 | 28.914 | 0.265 | Heptadecane | 90 | C_17_H_36_ | 240.47 |
| 54 | 29.049 | 0.816 | Hexadecane | 91 | C_16_H_34_ | 226.44 |
| 55 | 29.151 | 0.383 | Undecane, 2,4-dimethyl- | 80 | C_13_H_28_ | 184.36 |
| 56 | 29.302 | 0.353 | Eicosane | 93 | C_20_H_42_ | 282.55 |
| 57 | 29.555 | 0.226 | Heptacosane | 83 | C_27_H_56_ | 380.73 |
| 58 | 29.707 | 0.185 | Eicosane | 90 | C_20_H_42_ | 282.55 |
| 59 | 29.859 | 0.153 | Eicosane | 90 | C_20_H_42_ | 282.55 |
| 60 | 30.095 | 0.662 | Hexadecane, 3-methyl- | 87 | C_17_H_36_ | 240.47 |
| 61 | 30.180 | 0.771 | Heptadecane | 91 | C_17_H_36_ | 240.47 |
| 62 | 30.416 | 1.989 | Heneicosane | 90 | C_21_H_44_ | 296.57 |
| 63 | 30.568 | 0.610 | Tetracosane | 91 | C_24_H_50_ | 338.65 |
| 64 | 30.804 | 0.693 | Cycloheptasiloxane, tetradecamethyl- | 81 | C_14_H_42_O_7_Si_7_ | 519.08 |
| 65 | 31.107 | 11.563 | Phenol, 2,4-bis(1,1-dimethylethyl)- | 97 | C_14_H_22_O | 206.32 |
| 66 | 31.512 | 0.314 | Heptacosane | 64 | C_27_H_56_ | 380.73 |
| 67 | 31.850 | 0.449 | Octane, 5-ethyl-2-methyl- | 87 | C_11_H_24_ | 156.31 |
| 68 | 31.968 | 0.331 | Heptadecane, 2,6,10,15-tetramethyl- | 80 | C_21_H_44_ | 296.57 |
| 69 | 32.187 | 1.357 | Heptacosane | 80 | C_27_H_56_ | 380.73 |
| 70 | 32.575 | 0.440 | Eicosane | 90 | C_20_H_42_ | 282.55 |
| 71 | 32.913 | 0.184 | Eicosane | 91 | C_20_H_42_ | 282.55 |
| 72 | 33.031 | 0.240 | Eicosane | 96 | C_20_H_42_ | 282.55 |
| 73 | 33.317 | 0.219 | Hexadecane, 2,6,10,14-tetramethyl- | 90 | C_20_H_42_ | 282.55 |
| 74 | 33.419 | 0.102 | Eicosane | 89 | C_20_H_42_ | 282.55 |
| 75 | 33.807 | 0.104 | Eicosane | 90 | C_20_H_42_ | 282.55 |
| 76 | 34.144 | 0.117 | Eicosane | 90 | C_20_H_42_ | 282.55 |
| 77 | 34.228 | 0.140 | Phenol, 3,5-bis(1,1-dimethylethyl)- | 70 | C_14_H_22_O | 206.32 |
| 78 | 34.431 | 0.357 | Hexadecane | 97 | C_16_H_34_ | 226.44 |
| 79 | 34.718 | 0.345 | Pentadecane | 86 | C_15_H_32_ | 212.42 |
| 80 | 34.836 | 0.236 | Undecane, 3,5-dimethyl- | 76 | C_13_H_28_ | 184.36 |
| 81 | 34.988 | 0.223 | Eicosane | 91 | C_20_H_42_ | 282.55 |
| 82 | 35.224 | 0.150 | Hexadecane, 4-methyl- | 87 | C_17_H_36_ | 240.47 |
| 83 | 35.527 | 0.205 | 4-Amino-7-diethylamino-chromen-2-one | 64 | C_13_H_16_N_2_O_2_ | 232.28 |
| 84 | 35.645 | 0.126 | Eicosane | 89 | C_20_H_42_ | 282.55 |
| 85 | 35.764 | 0.553 | Eicosane | 91 | C_20_H_42_ | 282.55 |
| 86 | 36.286 | 0.672 | Pentadecane, 2,6,10-trimethyl- | 91 | C_18_H_38_ | 254.49 |
| 87 | 36.421 | 0.376 | Eicosane, 9-octyl- | 90 | C_28_H_58_ | 394.76 |
| 88 | 36.641 | 0.390 | Tetradecane | 90 | C_14_H_30_ | 198.39 |
| 89 | 36.759 | 0.482 | Dodecane, 2,6,11-trimethyl- | 93 | C_15_H_32_ | 212.41 |
| 90 | 37.079 | 0.228 | Eicosane | 92 | C_20_H_42_ | 282.55 |
| 91 | 37.214 | 0.601 | Octadecane, 9-ethyl-9-heptyl- | 47 | C_27_H_56_ | 380.73 |
| 92 | 37.383 | 0.552 | Eicosane | 91 | C_20_H_42_ | 282.55 |
| 93 | 37.653 | 0.750 | Heptadecane | 91 | C_17_H_36_ | 240.47 |
| 94 | 37.838 | 0.370 | Hexadecane | 91 | C_16_H_34_ | 226.44 |
| 95 | 38.041 | 0.810 | Nonadecane | 94 | C_19_H_40_ | 268.52 |
| 96 | 38.176 | 0.711 | Eicosane | 93 | C_20_H_42_ | 282.55 |
| 97 | 38.361 | 0.523 | Heneicosane | 91 | C_21_H_44_ | 296.57 |
| 98 | 38.496 | 1.707 | Heneicosane | 87 | C_21_H_44_ | 296.57 |
| 99 | 38.783 | 0.787 | Heneicosane | 91 | C_21_H_44_ | 296.57 |
| 100 | 39.019 | 0.520 | Dodecane, 4,6-dimethyl- | 94 | C_14_H_30_ | 198.39 |
| 101 | 39.391 | 0.621 | Hexadecane | 91 | C_16_H_34_ | 226.44 |
| 102 | 39.475 | 0.188 | Octadecane, 3-ethyl-5-(2-ethylbutyl)- | 80 | C_26_H_54_ | 366.71 |
| 103 | 39.660 | 0.361 | Hexadecane | 91 | C_16_H_34_ | 226.44 |
| 104 | 39.795 | 0.367 | Hexadecane | 91 | C_16_H_34_ | 226.44 |
| 105 | 40.032 | 1.440 | Eicosane | 90 | C_20_H_42_ | 282.55 |
| 106 | 40.352 | 0.524 | Eicosane | 91 | C_20_H_42_ | 282.55 |
| 107 | 40.487 | 0.330 | Eicosane | 91 | C_20_H_42_ | 282.55 |
| 108 | 40.639 | 0.217 | Hexadecane | 94 | C_16_H_34_ | 226.44 |
| 109 | 40.858 | 0.327 | Heptadecane | 91 | C_17_H_36_ | 240.47 |
| 110 | 41.179 | 0.162 | Eicosane | 64 | C_20_H_42_ | 282.55 |
| 111 | 41.617 | 0.309 | Octadecane | 98 | C_18_H_38_ | 254.49 |
| 112 | 41.803 | 0.363 | Eicosane | 96 | C_20_H_42_ | 282.55 |
| 113 | 41.955 | 0.195 | Eicosane | 87 | C_20_H_42_ | 282.55 |
| 114 | 42.14 | 0.113 | Eicosane | 87 | C_20_H_42_ | 282.55 |
| 115 | 42.343 | 0.139 | Eicosane | 91 | C_20_H_42_ | 282.55 |
| 116 | 42.478 | 0.178 | Heneicosane | 64 | C_21_H_44_ | 296.57 |
| 117 | 42.866 | 0.177 | Cyclononasiloxane, octadecamethyl- | 87 | C_18_H_54_O_9_Si_9_ | 666.39 |
| 118 | 43.254 | 1.240 | Heneicosane | 87 | C_21_H_44_ | 296.57 |
| 119 | 43.625 | 0.335 | Eicosane | 95 | C_20_H_42_ | 282.55 |
| 120 | 43.726 | 0.556 | Octadecane | 91 | C_18_H_38_ | 254.49 |
| 121 | 44.300 | 0.545 | 2-Butenoic acid, 2-methyl-, (E)- | 64 | C_5_H_8_O_2_ | 100.12 |
| 122 | 44.418 | 0.177 | Heneicosane | 90 | C_21_H_44_ | 296.57 |
| 123 | 44.569 | 0.469 | Heneicosane | 91 | C_21_H_44_ | 296.57 |
| 124 | 44.671 | 1.014 | Docosane | 91 | C_22_H_46_ | 310.6 |
| 125 | 44.958 | 1.054 | Nonadecane | 94 | C_19_H_40_ | 268.52 |
| 126 | 45.278 | 0.479 | Eicosane | 90 | C_20_H_42_ | 282.55 |
| 127 | 45.379 | 0.296 | Tridecane, 3-methyl- | 86 | C_14_H_30_ | 198.39 |
| 128 | 45.531 | 0.588 | Octacosane | 91 | C_28_H_58_ | 394.76 |
| 129 | 45.750 | 2.087 | Heneicosane | 90 | C_21_H_44_ | 296.57 |
| 130 | 45.987 | 0.715 | Heneicosane | 91 | C_21_H_44_ | 296.57 |
| 131 | 46.341 | 0.653 | Benzenepropanoic acid, 3,5-bis(1,1-dimethylethyl)-4-hydroxy-, methyl ester | 91 | C_18_H_28_O_3_ | 292.41 |
| 132 | 46.729 | 0.497 | Octacosane | 90 | C_28_H_58_ | 394.76 |
| 133 | 46.914 | 0.482 | Nonane, 5-butyl- | 89 | C_13_H_28_ | 184.36 |
| 134 | 47.100 | 1.371 | Eicosane | 91 | C_20_H_42_ | 282.55 |
| 135 | 47.336 | 0.411 | Heneicosane | 83 | C_21_H_44_ | 296.57 |
| 136 | 47.893 | 0.216 | Cyclodecasiloxane, eicosamethyl- | 46 | C_20_H_60_O_10_Si_10_ | 741.54 |
| 137 | 48.163 | 0.284 | Eicosane | 97 | C_20_H_42_ | 282.55 |
| 138 | 48.298 | 0.360 | Eicosane | 94 | C_20_H_42_ | 282.55 |
| 139 | 48.652 | 0.180 | Heneicosane | 91 | C_21_H_44_ | 296.57 |
| 140 | 48.787 | 0.138 | Eicosane | 90 | C_20_H_42_ | 282.55 |
| 141 | 49.361 | 0.300 | Tetracosane | 91 | C_24_H_50_ | 338.65 |
| 142 | 49.512 | 0.104 | Heneicosane | 90 | C_21_H_44_ | 296.57 |
| 143 | 49.580 | 0.135 | Eicosane | 91 | C_20_H_42_ | 282.55 |
| 144 | 50.018 | 0.604 | Heneicosane | 91 | C_21_H_44_ | 296.57 |
| 145 | 50.491 | 0.294 | Octadecane | 91 | C_18_H_38_ | 254.49 |
| 146 | 50.575 | 0.374 | Tetratriacontane | 81 | C_34_H_70_ | 478.92 |
| 147 | 50.879 | 0.342 | Eicosane | 93 | C_20_H_42_ | 282.55 |
| 148 | 51.081 | 0.517 | Heneicosane | 91 | C_21_H_44_ | 296.57 |
| 149 | 51.199 | 0.601 | Heneicosane | 91 | C_21_H_44_ | 296.57 |
| 150 | 51.284 | 0.617 | Pentacosane | 91 | C_25_H_52_ | 352.68 |
| 151 | 51.722 | 0.512 | Eicosane | 93 | C_20_H_42_ | 282.55 |
| 152 | 51.908 | 0.365 | Octacosane | 91 | C_28_H_58_ | 394.76 |
| 153 | 52.043 | 1.072 | Octadecanoic acid, methyl ester | 92 | C_19_H_38_O_2_ | 298.50 |
| 154 | 52.313 | 1.631 | Heneicosane | 90 | C_21_H_44_ | 296.57 |
| 155 | 52.515 | 0.493 | Octadecane, 9-ethyl-9-heptyl- | 59 | C_27_H_56_ | 380.73 |
| 156 | 53.106 | 0.728 | Octadecane, 2-methyl- | 91 | C_19_H_40_ | 268.52 |
| 157 | 53.291 | 0.592 | Eicosane | 94 | C_20_H_42_ | 282.55 |
| 158 | 53.527 | 1.123 | Heneicosane | 90 | C_21_H_44_ | 296.57 |
| 159 | 53.764 | 0.281 | Octacosane | 91 | C_28_H_58_ | 394.76 |
| 160 | 54.151 | 0.244 | Heneicosane | 91 | C_21_H_44_ | 296.57 |
| 161 | 54.219 | 0.224 | Heptadecane | 94 | C_17_H_36_ | 240.47 |
| 162 | 54.573 | 0.186 | Octacosane | 91 | C_28_H_58_ | 394.76 |
| 163 | 55.332 | 0.542 | Tetratriacontane | 91 | C_34_H_70_ | 478.92 |
| 164 | 55.518 | 0.095 | Eicosane | 95 | C_20_H_42_ | 282.55 |
| 165 | 55.670 | 0.173 | Hexadecane, 1-iodo- | 91 | C_16_H_33_I | 352.34 |
| 166 | 56.041 | 0.502 | 1-Cyclohexyldimethylsilyloxy-3,5-dimethylbenzene | 32 | C_16_H_26_OSi | 262.46 |
| 167 | 56.176 | 0.239 | Eicosane | 94 | C_20_H_42_ | 282.55 |
| 168 | 56.733 | 0.333 | Cyclononasiloxane, octadecamethyl- | 10 | C_18_H_54_O_9_Si_9_ | 666.39 |
| 169 | 56.851 | 0.989 | Dodecane, 2,6,11-trimethyl- | 53 | C_15_H_32_ | 212.41 |
| 170 | 57.154 | 0.485 | Eicosane | 93 | C_20_H_42_ | 282.55 |
| 171 | 57.256 | 0.245 | Eicosane | 93 | C_20_H_42_ | 282.55 |
| 172 | 57.357 | 0.428 | Heptadecane, 3-methyl- | 93 | C_18_H_38_ | 254.49 |
| 173 | 57.509 | 0.398 | Heptadecane, 3-methyl- | 95 | C_18_H_38_ | 254.49 |
| 174 | 57.677 | 0.537 | Heptadecane, 3-methyl- | 95 | C_18_H_38_ | 254.49 |
| 175 | 57.998 | 1.406 | 3,6-Dioxa-2,7-disilaoctane, 2,2,7,7-tetramethyl-4,5-diphenyl- | 47 | C_20_H_30_O_2_Si_2_ | 358.62 |
| 176 | 58.183 | 0.869 | Octacosane | 91 | C_28_H_58_ | 394.76 |
| 177 | 58.318 | 0.624 | Octadecane | 95 | C_18_H_38_ | 254.49 |
| 178 | 58.504 | 0.602 | Pentacosane | 91 | C_25_H_52_ | 352.68 |
| 179 | 58.993 | 0.366 | Hentriacontane | 91 | C_31_H_64_ | 436.84 |
| 180 | 59.162 | 0.378 | Octadecane | 95 | C_18_H_38_ | 254.49 |
| 181 | 59.331 | 0.673 | Pentacosane | 87 | C_25_H_52_ | 352.68 |
| 182 | 59.432 | 0.517 | Tetratriacontane | 91 | C_34_H_70_ | 478.92 |
| 183 | 59.634 | 0.515 | Octadecane | 93 | C_18_H_38_ | 254.49 |
| 184 | 60.646 | 0.368 | Octadecane, 3-ethyl-5-(2-ethylbutyl)- | 52 | C_26_H_54_ | 366.71 |
| 185 | 61.810 | 0.247 | Eicosane | 95 | C_20_H_42_ | 282.55 |
| 186 | 62.232 | 0.166 | Eicosane | 94 | C_20_H_42_ | 282.55 |
| 187 | 62.536 | 0.356 | Decane, 3,8-dimethyl- | 92 | C_12_H_26_ | 170.33 |
| 188 | 63.143 | 0.503 | Heptadecane, 3-methyl- | 94 | C_18_H_38_ | 254.49 |
| 189 | 63.430 | 0.329 | Heptadecane, 3-methyl- | 94 | C_18_H_38_ | 254.49 |
| 190 | 64.088 | 0.261 | Octacosane | 83 | C_28_H_58_ | 394.76 |
| 191 | 64.425 | 0.617 | Hexacosane | 91 | C_28_H_58_ | 394.76 |
| 192 | 64.695 | 0.235 | Hentriacontane | 91 | C_31_H_64_ | 436.84 |
